# The Role of Sodium-Glucose Cotransporter-2 Inhibitors in Adults With Transthyretin Cardiac Amyloidosis: A Single-Center Retrospective Cohort Study

**DOI:** 10.7759/cureus.72725

**Published:** 2024-10-30

**Authors:** Ashwin Pillai, Sana Riaz, Sabeena Arora, Abhishek Jaiswal

**Affiliations:** 1 Medicine, University of Connecticut Health, Farmington, USA; 2 Cardiology, Texas Heart Institute at Baylor St. Luke's Medical Center, Houston, USA; 3 Cardiology, Hartford Hospital, Hartford, USA

**Keywords:** attr cardiac, cardiac amyloidosis, guideline directed medical therapy, guideline-directed medical therapy (gdmt), heart failure, heart failure cardiac amyloidosis, sglt2-inhibitors, sglt2 inhibitors and heart failure, sodium-glucose cotransporter-2 (sglt2) inhibitors, transthyretin cardiac amyloidosis

## Abstract

Background: Cardiac amyloidosis (CA) is an infiltrative cardiomyopathy with limited treatment options. Barring mineralocorticoid receptor antagonists, most classes of guideline-directed medical therapy including renin-angiotensin-aldosterone inhibitors and beta blockers are avoided in CA due to intolerance and the risk of potentiating orthostatic hypotension. Few studies have explored the safety and utility of sodium-glucose cotransporter-2 inhibitors (SGLT2is) in CA. These agents have demonstrated benefit in most heart failure phenotypes, and individuals with CA may benefit from treatment with them. We report a single-center experience demonstrating the safety of SGLT2is in patients with cardiac amyloidosis.

Methods: We conducted a retrospective study including patients with CA from January 2020 to March 2023 who were treated with empagliflozin at doses of 10 mg or 25 mg daily or dapagliflozin at a dose of 10 mg daily. Patients were monitored clinically for the development of adverse effects including urinary tract infections and orthostatic hypotension as well as through laboratory monitoring for acute kidney injury and for changes in microalbuminuria.

Results: Our cohort comprised 53 patients with cardiac amyloidosis treated with an SGLT2i, 38 (72%) of whom were male, with a mean participant age of 81±8 years and left ventricular ejection fraction of 50%. Urinary tract infections were observed in three participants (6%). Acute kidney injury occurred in two participants. No participant experienced orthostatic hypotension. There was a trend toward improved functional status, reduced hospitalizations (decrease from median 1 to 0.6 per patient-year, p=0.18), and microalbuminuria (mean albumin:creatinine ratio of 53.8 mg/g improved to 29.2 mg/g, p=0.32), although these differences were not statistically significant.

Conclusions: Within our cohort of individuals with cardiac amyloidosis, SGLT2i therapy appeared to be well-tolerated and may have clinical benefit in the form of reduced hospitalizations and improvements in microalbuminuria. Notably, the differences in outcomes were not statistically significant. By demonstrating safety and tolerance, we hope to stimulate prospective randomized studies that may definitively explore the utility of SGLT2i therapy in enhancing clinical outcomes.

## Introduction

Cardiac amyloidosis is an infiltrative cardiomyopathy caused by the deposition of misfolded protein aggregates resulting in compromised cardiac function. It can manifest as heart failure with preserved (HFpEF), mildly reduced (HFmrEF), or reduced ejection fraction (HFrEF). Its prevalence in the general population is estimated to be one in 6,000 (~0.01%), increasing to approximately 1% among patients with heart failure, 13% in patients with HFpEF, and 20% among a subset of patients with a myocardial thickness exceeding 14 mm [1,2]. However, this may be an underestimation of the true disease burden. With improvements in non-invasive diagnostic modalities, the measured prevalence is expected to increase and gradually approximate the true burden of disease. Recent studies have documented an increase in prevalence rate from 8 to 17 per 100,000 person-years and incidence rate from 18 to 55 per 100,000 person-years between 2000 and 2012, with the increase in the rate of detection accelerating after 2006 [3].

Medical treatment of heart failure (HF) in transthyretin cardiac amyloidosis (ATTR-CA) has been limited primarily to loop diuretics and mineralocorticoid receptor antagonists due to intolerance of other agents with proven benefits, including cardio-selective beta blockers, renin-angiotensin-aldosterone system inhibitors, and angiotensin receptor neprilysin inhibitors that may potentiate the risk for orthostatic hypotension. Sodium-glucose cotransporter-2 inhibitors (SGLT2is) have significantly improved outcomes in patients with HF regardless of ejection fraction, concurrent diabetes, or renal disease. However, data and clinical experience in ATTR-CA-associated HF is limited [4-6]. The direct cardioprotective, antifibrotic, anti-inflammatory, and diuretic effects with minimal deleterious hemodynamic effects make SGLT2 inhibitors a promising agent to treat ATTR-CA-associated HF. We explored the long-term safety and tolerability of SGLT2 inhibitors in ATTR-CA-associated HF patients enrolled in our cardiac amyloidosis management program (CAMP). This article was previously presented as a meeting abstract at the 2023 Annual Scientific Meeting of the Heart Failure Society of America on October 7, 2023 [7].

## Materials and methods

Study design

We conducted a retrospective review of patients enrolled in the Hartford Hospital Cardiac Amyloidosis Program (initiated in 2019) reviewing records from January 2020 to March 2023 [8]. The Cardiac Amyloidosis Program has enrolled 319 patients since inception.

Institutional review board (IRB) approval

Institutional review board (IRB) exemption was granted by the Hartford Healthcare Institutional Review Board (IRB#: HHC-2022-0303, reference number: 032512) with waiver of informed consent and Health Insurance Portability and Accountability Act (HIPAA) Authorization for Research under CFR164.512(i)(2)(ii).

Participants

We included all serially enrolled participants in the Cardiac Amyloidosis Program aged 18 years and above, independent of race, ethnicity, and gender, who received a confirmed diagnosis of cardiac amyloidosis. Exclusions were limited to pregnant individuals or patients under the age of 18 years.

Definitions and measurements

Acute Kidney Injury

Acute kidney injury defined by the Kidney Disease Improving Global Outcomes (KDIGO) as an acute rise in serum creatinine by 0.3 mg/dl within 48 hours or increase by greater than 50% from baseline within seven days of initiating the SGLT2i.

Cardiac Amyloidosis

A diagnosis of cardiac amyloidosis was made in an individual with heart failure, negative serum and urine immunofixation and either a positive Pyrophosphate (PYP) scintigraphy scan, diagnostic cardiac magnetic resonance imaging (MRI), or an endomyocardial biopsy.

Proteinuria

Proteinuria defined as protein:creatinine ratio >114 mg/g per internal laboratory assay standards.

Urinary Tract Infections

Urinary tract infections diagnosed by urinalysis and culture. A positive study was defined as urinalysis demonstrating >10 WBCs per high power frame on microscopy with or without leukocyte esterase or nitrates and with culture demonstrating a single organism with a colony count >105 colony-forming units per ml [9].

Data Sources

Data was obtained by reviewing the Hartford Hospital electronic medical record system.

Sample Size and Sample Size Justification

We included a total of 53 patients in the study. All patients enrolled in the cardiac amyloidosis program were serially included. Given the descriptive aim of the study, a post hoc power analysis was not conducted.

Methodology

Records for all patients enrolled in the cardiac amyloidosis program were reviewed and patients who were treated with empagliflozin at doses of 10 mg or 25 mg daily or dapagliflozin at a dose of 10 mg daily were included. Data extracted included patient demographics, comorbidities, laboratory records, clinical symptoms, imaging findings, and medication lists. Comorbidities were qualitatively enumerated as present or absent; clinical symptoms of heart failure were semi-quantitatively graded in accordance with the New York Heart Association (NYHA) classification. Once identified, records of patients who received SGLT2i were longitudinally reviewed and data concerning clinical outcomes were extracted and recorded. Patients were monitored clinically for the development of adverse effects including urinary tract infections and orthostatic hypotension as well as through laboratory monitoring for acute kidney injury and for changes in microalbuminuria.

Statistical Analysis

For the description of cohort characteristics, we used mean ± standard deviation or median (interquartile range (IQR)), depending on whether the data was normally distributed, as measures of the central tendency of the data. The Shapiro-Wilk test was used to determine normalcy. Mann Whitney’s U-test was used to compare non-parametric data, and student’s T-test to compare normally distributed data across two arms. For categorical data, we used Fisher’s exact test to compare proportional event outcomes. Data was manually abstracted and analyzed using Microsoft Excel 2016 (Microsoft Corporation Inc., Redmond, United States of America).

Funding

This research did not receive any specific grant from funding agencies in the public, commercial, or not-for-profit sectors. The study was investigator-initiated.

## Results

**Figure 1:**
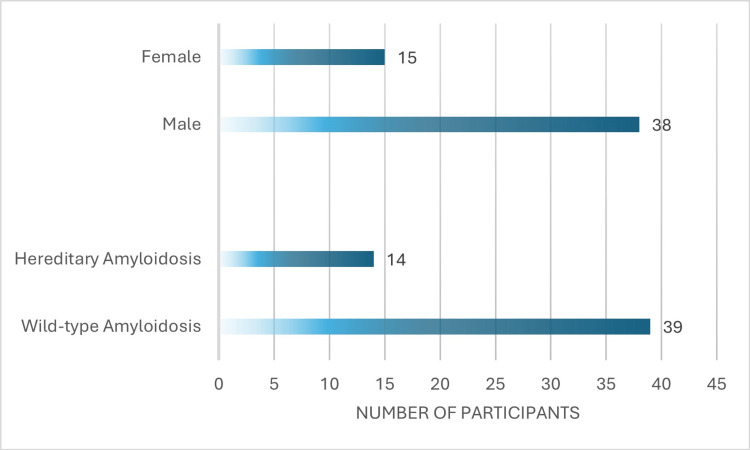
Cohort demographic characteristics

We included a total of 53 patients with ATTR cardiac amyloidosis (CA) in the analysis. Most participants had wild-type ATTR-CA (n=39, 74%) with 14 (26%) participants screening positive for genetic mutations associated with hereditary ATTR-CA. Our cohort was predominantly male with 38 (72%) male participants and 15 (28%) female participants. The mean age of participants was 81±8 years. Cohort demographics are represented in Figure 1.

**Figure 2:**
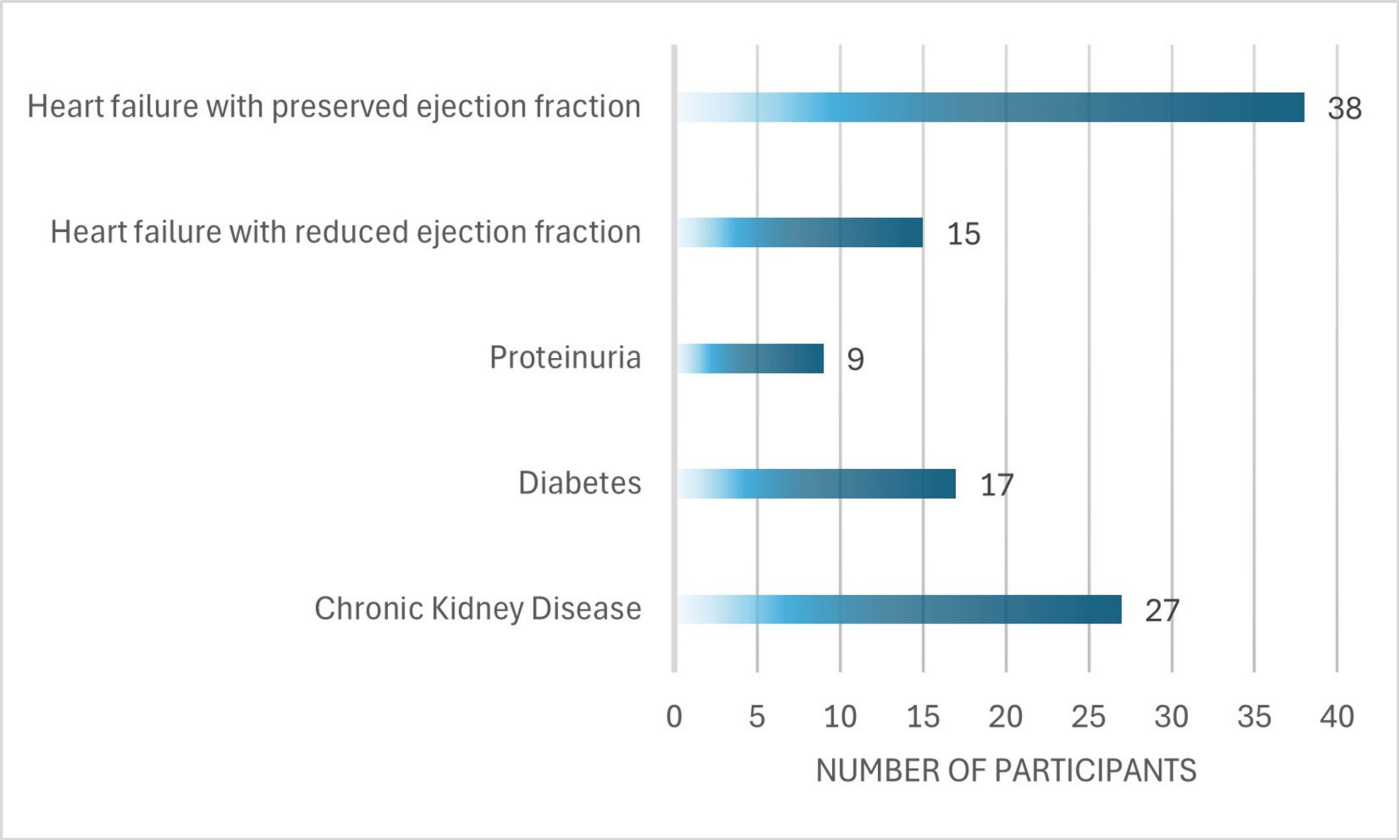
Participant comorbidities within the cohort

Within our cohort, 27 participants (51%) had chronic kidney disease with an estimated glomerular filtration rate (eGFR) <60 ml/min/m^2^ with a mean eGFR of 58±18 ml/min/m^2^, 17 (32%) had diabetes mellitus (DM). Proteinuria was assessed for 18 participants and was noted in nine (50%) individuals. The median left ventricular ejection fraction (LVEF) was 50% (IQR: 36, 55). An LVEF <40% was noted in 15 (28%) participants. Participant comorbidities are represented in Figure 2.

**Figure 3:**
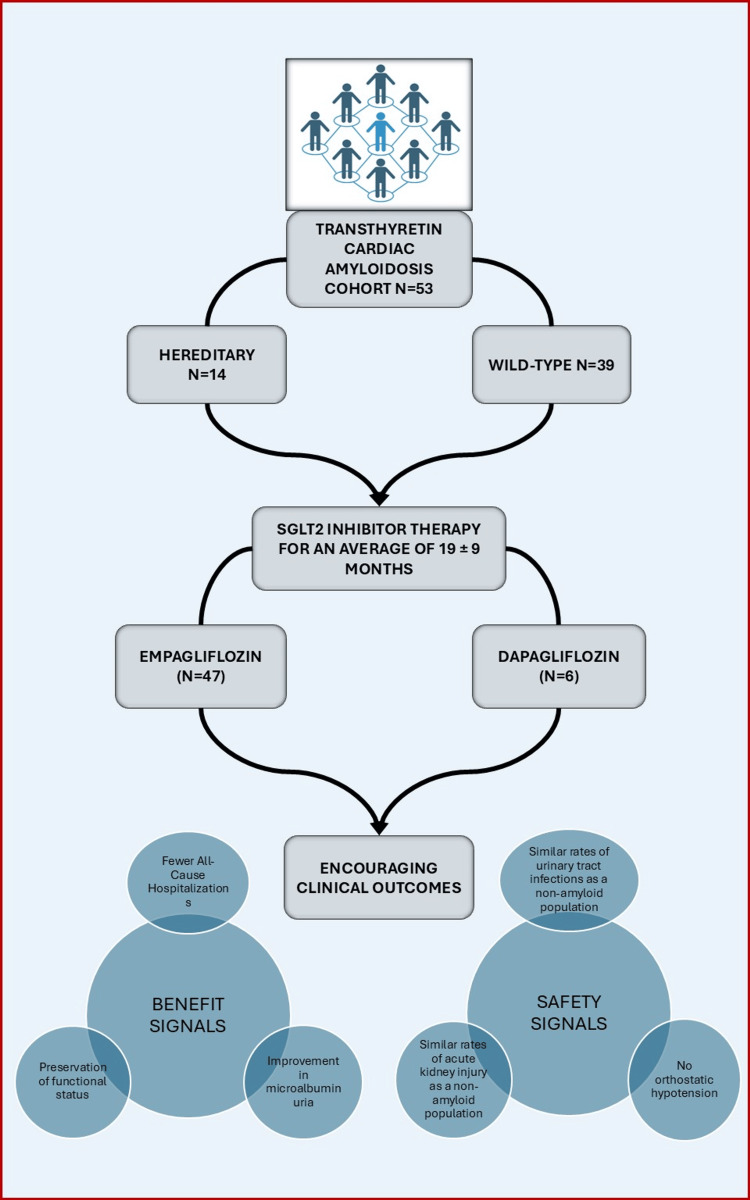
Study design, participant characteristics, and outcomes SGLT2: sodium-glucose cotransporter-2. Figure credits: Ashwin Pillai.

Most participants received empagliflozin (n=47, 89%), and six (11%) participants received dapagliflozin. None of the participants received canagliflozin. The mean duration of SGLT2i therapy was 19±9 months. While receiving treatment with an SGLT2i, most patients received at least one other class of medication included under guideline-directed medical therapy (GDMT), the most frequently prescribed agent being a mineralocorticoid receptor antagonist in 46 (87%) participants. One patient required inotropic therapy that was initiated concurrently with the SGLT2i. Cohort outcomes are summarized in Table 1 and Figure 3.

**Table 1:** Characteristics and clinical outcomes of patients with heart failure and transthyretin cardiac amyloidosis treated with SGLT2 inhibitors SD: standard deviation; SGLT2: sodium-glucose cotransporter-2.

Outcomes	Result
Duration of follow-up in months for the cohort (mean ± SD)	19 ± 9
Duration of follow-up in months for empagliflozin (mean ± SD)	22 ± 7
Duration of follow-up in months for dapagliflozin (mean ± SD)	18 ± 9
Adverse reactions	
Urinary tract infection (n)	3 (6%)
Acute kidney injury (n)	2 (3.8%)
Drug discontinuation (n)	3
Complicated urinary tract infection (n)	1
Discontinuation by the patient (n)	1
Transition to Hospice Care (n)	1

Following the initiation of an SGLT2i, the mean serum creatine remained unaffected (1.4 ± 0.6 mg/dl pre-SGLT2i vs. 1.5 ± 0.6 mg/dl post-SGLT2i, p=0.5). Acute kidney injury occurred in two participants (3.8%). Urinary tract infections occurred in three participants (6%). New orthostatic hypotension was not noted in any patient while receiving an SGLT2i. The SGLT2i was discontinued in three patients (6%): one patient each for a complicated urinary tract infection (UTI), transition to hospice, or patient-initiated discontinuation. Functional status was preserved over the duration of follow-up with a tendency toward improvement from New York Heart Association functional class III to class II (p=0.7). This trend is represented in Table 2.

**Table 2:** Functional status outcomes for the cohort NYHA: New York Heart Association; SGLT2i: sodium-glucose cotransporter-2 inhibitor. * not statistically significant. A p-value <0.05 was considered statistically significant.

NYHA class					P-value
	Class I (n)	Class II (n)	Class III (n)	Class IV (n)	0.73^*^
Pre-SGLT2i therapy	5	10	31	7	
On SGLT2i therapy	5	13	25	9	

While on treatment with the SGLT2i, there was a statistically non-significant decrease in the number of hospitalizations. The median (IQR) annual hospitalization rates were 1 (IQR: 0, 2) pre-SGLT2i versus 0.6 (IQR: 0, 1.2) per patient-year, p=0.18, with a total of 94 all-cause hospitalizations. A similar tendency toward improvement in microalbuminuria was seen in the six patients for whom microalbuminuria trends were available, improving from a mean of albumin:creatinine ratio of 53.8 mg/g to 29.2 mg/g (p=0.32) after a median follow-up of 12 months (IQR: 8, 18) (Table 1). There were no deaths during the duration of follow-up.

## Discussion

Cardiac amyloidosis is a clinical entity characterized by the deposition of misfolded protein aggregates resulting in compromised cardiac function. Two precursor proteins have been implicated in the pathogenesis of this infiltrative process: amyloid light chains (causing AL amyloidosis) and amyloid transthyretin (causing ATTR amyloidosis). AL amyloidosis is caused by the overproduction of kappa or lambda immunoglobulin light chains by plasma cells [10]. Transthyretin cardiac amyloidosis (ATTR-CA) can occur either de novo typically in association with aging (wild-type or wt-ATTR) or can be consequent to an inherited genetic mutation (hereditary or h-ATTR) [11]. Conventionally, cardiac amyloidosis has been a challenging entity to treat, and clinical outcomes have been suboptimal [12]. Conventional guideline-directed medical therapy is generally considered less beneficial or potentially harmful in CA [13]. However, the ATTR-ACT (2018), APOLLO-B (2023), and ATTRIBUTE-CM (2024) trials have been promising, demonstrating the utility of the disease-modifying agents tafamidis, patisiran, and acoramidis, respectively [14-16]. Moreover, recent studies have demonstrated the safety and utility of conventional guideline-directed medical therapy in heart failure phenotypes previously considered less likely to benefit from such treatment options, prompting investigators to revisit the potential utility of medical therapy in CA [17,18]. SGLT2 inhibitors decrease mortality and morbidity in patients with heart failure notwithstanding their phenotype and are generally considered safe regardless of the etiology and these benefits are expected early in the course of treatment, often as early as within 8-10 months [19,20]. However, data supporting the safe use of SGLT2i in cardiac amyloidosis has, until recently, been scarce [4]. Reports continue to emerge supporting the safety and potentially positive clinical impact of SGLT2i use in patients with ATTR-CA-associated HF [5,6,20].

Our study supports the safety and tolerability of SGLT2 inhibitors in a relatively large series of patients with ATTR-CA-associated symptomatic HF across ejection fractions, NYHA functional classes, and renal function. Strengths of this study include the use of multiple SGLT2i agents including empagliflozin and dapagliflozin, thus affording a degree of generalizability to a drug class rather than a single agent. Our cohort did not observe adverse outcomes with SGLT2i therapy compared with standard therapy. Adverse events occurred at rates comparable to those reported in current literature, the incident rate of 6% for UTIs observed in our cohort is within the expected range of 4%-9% while on SGLT2i therapy [21]. Further, our cohort comprised a relatively unwell population, over half of whom had chronic kidney disease. Proteinuria was also present in half of those assessed for it, conferring an even higher risk of adverse cardiovascular outcomes [22]. Even in this high-risk cohort, we observed a promising tendency toward improvement in all-cause hospitalizations and microalbuminuria, although the results were not statistically significant given our limited sample sizes. Additionally, our cohort generally demonstrated a preserved functional status across the duration of follow-up with a numerical, but statistically insignificant, increase in the number of individuals with NYHA class II symptoms and a decrease in the number of individuals with class III symptoms. We may cautiously interpret these findings as suggestive of the benefit that SGLT2i therapy may offer in terms of preserved functional status, which may contribute to improved quality of life, although prospective studies powered to detect such differences will be required to assess such effects conclusively. The low rates of adverse events demonstrating tolerance and the preservation of NYHA functional class are consistent with current literature (Zampieri et al., 2022, and Dobner et al., 2023) [5,6].

It is important to interpret the findings of our study in the context of its limitations. Due to the relatively limited sample size of our single-center study, our findings may not be generalizable to individuals of all ethnicities. The retrospective nature of the study, the limited sample size, and relatively low event rate also limit the external validity of the differences in clinical outcomes noted with SGLT2i therapy, which were not statistically different. Given that a minority of our cohort were assessed for proteinuria and microalbuminuria (which was assessed at the discretion of the provider), the findings are susceptible to selection bias.

However, our report does show that SGLT2i therapy might provide another safe avenue for the clinical management of ATTR-CA-associated HF. Current therapies approved for ATTR-CA can help prevent progression but are unable to truly reverse the disease process. Although transplant remains a viable option for patients with end-stage heart failure due to ATTR-CA, waitlist times are often longer compared to those for both patients with AL CA and for the general population [23,24]. Furthermore, as the global demographic ages, the prevalence of heart failure will continue to rise and, with it, so will the incidence of cardiac amyloidosis [25]. This is likely to lead to an increased need for heart transplants. The aging demographic, however, poses yet another concern, the concern about the rising age of donor hearts, a known contributor to suboptimal transplant outcomes [26]. The use of durable mechanical circulatory supports as an alternative to transplant is also riddled with challenges, with patients with CA often requiring biventricular support [27].

Given these limitations to definitive therapy for end-stage heart failure, further studies exploring the utility of conventional guideline-directed medical therapy are the need of the hour. As SGLT2 inhibitors improve morbidity and survival in HF patients by direct cardioprotective, antifibrotic, and anti-inflammatory effects and ATTR-CA is underdiagnosed in the HF population, it remains a possibility that SGLT2 inhibitors are well tolerated, improve morbidity as well as survival, and facilitate reverse remodeling in ATTR-CA [28].

## Conclusions

Our single-center study demonstrated the long-term safety of SGLT2i therapy in the treatment of heart failure due to cardiac amyloidosis. Patients treated with SGLT2is did not experience higher-than-expected rates of treatment discontinuation or treatment-limiting adverse effects such as acute kidney injury, orthostatic hypotension, or urinary tract infections. Although our study suggested potentially improved outcomes in the form of preserved functional status, reduced hospitalizations, and improvements in microalbuminuria, these differences were not statistically significant and limited by the sample size and low event rate. By demonstrating safety and tolerance, we hope to stimulate multi-center, prospective, randomized trials that may definitively explore the utility of SGLT2i therapy in enhancing clinical outcomes in cardiac amyloidosis.
